# Assessing Quality of Falls Assessments in Belvedere and Rougemont Wards at Franklyn Community Hospital

**DOI:** 10.1192/bjo.2026.11716

**Published:** 2026-06-30

**Authors:** Reham Abdelsalam, Benjamin Geers, Craig Chandler, Sam Wildgoose, Lewis Powell

**Affiliations:** Franklyn Hospital, Exeter, United Kingdom

## Abstract

**Aims:**

The aims were set to ensure that the falls assessments documentation are emphasising patients who had an inpatient fall to be assessed by a clinician within 12 hours of the fall as well as including NICE guidelines for head injuries, reviewing medication chart and documenting treatment escalation plans of the patients assessed.

**Methods:**

We collected data from 23 reported falls on our DATIX system between August 2025 till November 2025. We searched our patients’ online system for the details of the fall, whether seen by a clinician within 12 hours, clearly documenting head injuries and NICE head injury guideline plans, medication reviews as well as treatment escalation plans.

**Results:**

Patients on Rougemont ward had only 58% of their medications reviewed when a falls assessment was carried out. Whereas patients on Belvedere ward had 52% of the medication reviewed. In addition, Rougemont ward had 0% of treatment escalation plans reviewed whereas Belvedere ward had 43% of the falls with clear documentation on the treatment escalation plan.

**Conclusion:**

The audit offered in-depth research into the falls assessments happening on both Rougemont and Belvedere wards at Franklyn Hospital. The data showed good overall percentages in patient’s being assessed 12 hours post-fall and at least 52-58% of falls of patients had medications reviewing. However, TEP review and documentation still requires improvement as well as clear documentation of NICE head injury guidelines in the management plan. These could be improved through a more standardised proforma including these essential details and re-auditing to see the change. Ultimately, improving patient’s falls assessments and patient’s care.

